# Meta-analysis of Ginkgo biloba extract EGb 761 in the treatment of mild dementia

**DOI:** 10.1192/j.eurpsy.2025.1749

**Published:** 2025-08-26

**Authors:** M. Riepe, M. Burkart

**Affiliations:** 1 Division of Geriatric Psychiatry, Ulm University, Ulm; 2 Global Medical Affairs, Dr. Schwabe Holding SE & Co. KG, Karlsruhe, Germany

## Abstract

**Introduction:**

Even mild forms of dementia have a detrimental effect on memory and activities of daily living, and cause distress to patients and their families. As the disease progresses, the impairment of patients and the burden on their carers increases over time. Thus, there is a need for effective, safe and well-tolerated treatments that can be initiated at the earliest stages.

**Objectives:**

A meta-analysis of pooled patient subgroup data from randomised clinical trials was conducted to assess the treatment effects of Ginkgo biloba extract EGb 761 in patients with mild dementia.

**Methods:**

The studies included in this meta-analysis were selected from a previous systematic review (von Gunten *et al*. World J Biol Psychiatry 2016, 17(8),622-633). They enrolled patients with mild dementia (total score 9-15 on the SKT Short Cognitive Performance Test, SKT) (Lehfeld and Erzigkeit, Int Psychogeriatr 1997, 9(Suppl 1), 115-21) with probable Alzheimer’s disease, probable vascular dementia, or possible Alzheimer’s disease with cerebrovascular disease, respectively. Outcome measures were cognition, activities of daily living, global clinical assessment and quality of life.

**Results:**

From four eligible trials data of 782 patients with mild dementia were included in the meta-analysis. The analysis demonstrated that treatment with 240 mg EGb 761 daily was significantly superior to placebo in cognition (p=0.04), global assessment (p=0.01), activities of daily living (p=0.01) and quality of life (p=0.02) with medium to large standardised effects. Adverse events were similarly frequent in patients treated with EGb 761 and placebo (p=0.66).

**Image:**

**Figure:**
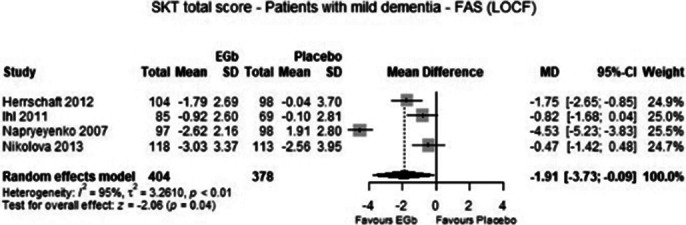

**Image 2:**

**Figure:**
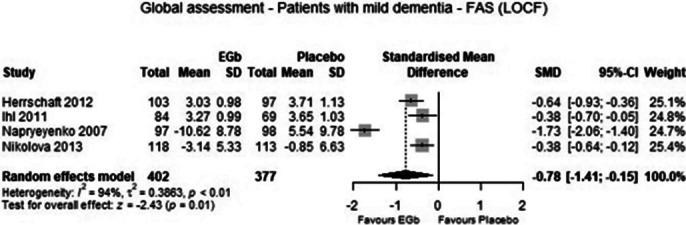

**Image 3:**

**Figure:**
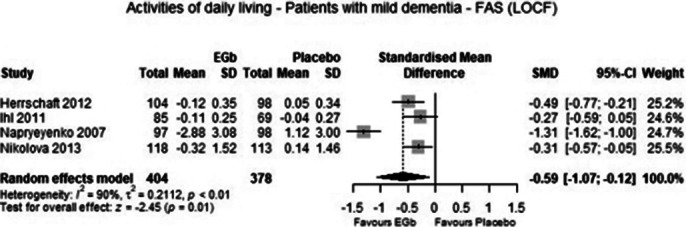

**Conclusions:**

The meta-analysis shows that EGb 761 has beneficial effects on cognition, activities of daily living, global assessment and quality of life in patients with mild dementia.

**Disclosure of Interest:**

M. Riepe Speakers bureau of: Dr. Willmar Schwabe GmbH & Co. KG, M. Burkart Employee of: Dr. Schwabe Holding SE & Co. KG

